# Comparing mental and physical health of U.S. veterans by VA healthcare use: implications for generalizability of research in the VA electronic health records

**DOI:** 10.1186/s12913-022-08899-y

**Published:** 2022-12-09

**Authors:** David S. Fink, Malka Stohl, Zachary L. Mannes, Dvora Shmulewitz, Melanie Wall, Sarah Gutkind, Mark Olfson, Jaimie Gradus, Salomeh Keyhani, Charles Maynard, Katherine M. Keyes, Scott Sherman, Silvia Martins, Andrew J. Saxon, Deborah S. Hasin

**Affiliations:** 1 New York State Psychiatric Institute, New York, NY USA; 2 Columbia University Mailman School of Public Health, New York, NY USA; 3 Boston University School of Public Health, Boston, MA USA; 4 Veteran Affairs, San Francisco, VA USA; 5 University of California, San Francisco, CA USA; 6 Veteran Affairs, Puget Sound Health Care System, Seattle, WA USA; 7 University of Washington, Seattle, WA USA; 8 New York University, New York, NY USA; 9 Department of Psychiatry, Columbia University Medical Center, 1051 Riverside Dr., Unit 123, New York, NY 10032 USA

**Keywords:** Generalizability, Big data, Electronic health records, United States Department of Veterans Affairs, Veterans health

## Abstract

**Objective:**

The Department of Veterans Affairs’ (VA) electronic health records (EHR) offer a rich source of big data to study medical and health care questions, but patient eligibility and preferences may limit generalizability of findings. We therefore examined the representativeness of VA veterans by comparing veterans using VA healthcare services to those who do not.

**Methods:**

We analyzed data on 3051 veteran participants age ≥ 18 years in the 2019 National Health Interview Survey. Weighted logistic regression was used to model participant characteristics, health conditions, pain, and self-reported health by past year VA healthcare use and generate predicted marginal prevalences, which were used to calculate Cohen’s *d* of group differences in absolute risk by past-year VA healthcare use.

**Results:**

Among veterans, 30.4% had past-year VA healthcare use. Veterans with lower income and members of racial/ethnic minority groups were more likely to report past-year VA healthcare use. Health conditions overrepresented in past-year VA healthcare users included chronic medical conditions (80.6% vs. 69.4%, *d = 0.36)*, pain (78.9% vs. 65.9%; *d* = 0.35), mental distress (11.6% vs. 5.9%; *d* = 0.47), anxiety (10.8% vs. 4.1%; *d* = 0.67), and fair/poor self-reported health (27.9% vs. 18.0%; *d* = 0.40).

**Conclusions:**

Heterogeneity in veteran sociodemographic and health characteristics was observed by past-year VA healthcare use. Researchers working with VA EHR data should consider how the patient selection process may relate to the exposures and outcomes under study. Statistical reweighting may be needed to generalize risk estimates from the VA EHR data to the overall veteran population.

**Supplementary Information:**

The online version contains supplementary material available at 10.1186/s12913-022-08899-y.

## Introduction

The Veterans Affairs (VA) is the largest integrated health care delivery system in the United States (US), providing care to over 6 million eligible veterans each year within 1200 geographically dispersed health care facilities [1–3]. With the size and scope of the VA health care delivery system comes a vast quantity of data on diagnoses, medication history, and sociodemographics, as well as provider and facility characteristics—all of which are stored in the VA electronic health records (EHR). Integration of data from several outside sources into the VA EHR (e.g., data for patients receiving care through the VA’s Community Care Program, Medicare data for patients aged ≥65) provide a nearly complete record of health care at both the patient- and organizational-level and an ideal data source for studying clinically important questions in veterans health. However, a major concern for the generalizability of EHR-based studies is selection bias, which is a systematic error of effect estimates introduced if the association between exposure and disease differs between those who contribute data to an EHR and those who do not [4–6]. Risk of selection bias is particularly high in cases when a subset of a population with a specific risk profile is strongly underrepresented in the study sample. As such, the VA beneficiary characteristics derived from the EHR raise concerns about selection bias and the generalizability of VA EHR-based research to the larger US veteran population. Nevertheless, the vast quantity of health-related data captured in the VA’s EHR represents an important resource for conducting health services research when properly interpreted.

The US Department of Veteran Affairs is a cabinet-level department tasked with providing services and benefits to US military veterans. The VA EHR data offer a critical tool for achieving these aims: they can be used to inform the development of prevention strategies tailored to the unique characteristics and needs of US veterans and to evaluate the efficacy of clinical and public health interventions. For example, veteran are recognized as a population at elevated risk of suicide [7, 8] and suicide prevention is an explicit focus of care improvement in the VA health system [9–11]. Recent research has used VA EHR data and machine learning technology to predict suicidal behavior in VA patients [12–14]. Using the information generated from this research, the VHA began national implementation of the Recovery Engagement and Coordination for Health-Veterans Enhanced Treatment (REACH VET) program, which applied the algorithm to identify patients in the highest suicide risk [12]. Using VA EHR data, subsequent studies evaluated the impact of the REACH VET program, finding that it was associated with greater treatment engagement and fewer mental health admissions, emergency department visits, and suicide attempts [15].

In addition to providing useful knowledge for improving the public health and medical care of US veterans, VA EHR data can be used to study the efficacy of clinical interventions, which can then be used to improve clinical care in the general US population. For example, although randomized controlled trials are generally considered the gold standard to determine the efficacy of medications and health care interventions, they are expensive, time-prohibitive, difficult to implement, and ill-suited for the study of high-risk interventions, rare outcomes, and the consequences of harmful exposures (e.g., exposure to potentially traumatic events) [16, 17]. Electronic health record data can provide information about the efficacy of health interventions in instances when randomized controlled trials are unfeasible or undesirable. For example, EHR data have been used to provide rapid results during the COVID-19 pandemic about the potential protective effect of some antihistamines on risk of SARS-CoV-2 infection [18]. However, demonstrating the efficacy of a treatment in one study sample (e.g., VA patients) does not necessarily provide evidence of its efficacy in other populations, whether that is US veterans in general or the total US adult population. For example, caution is warranted when interpreting results from EHR based studies due to selection bias. One particular type of selection bias of concern that is present in EHR based studies is called collider bias, which arises from the exposure of interest being associated with the likelihood of being observed and can result in spurious associations when none exists [19]. As such, no matter how rigorous or carefully executed an EHR-based research study, the results depend on the setting in which they were derived (e.g., VA patient population), and often depend on factors that might be constant within the studied population but different elsewhere. Because any given association between an exposure and an outcome will vary across settings and populations as a function of how different the study sample (e.g., VA patient population) and the target population (e.g., non-VA veteran population) are from one another, information on the distribution of covariates in the VA patient population and non-VA veteran population must be considered to use the knowledge generated from research conducted in VA EHR data to inform policy for populations outside the VA patient population.

Users of VA healthcare represent a population with greater physical, mental, and social challenges than the general US adult population [20–22] as well as the overall US veteran population [23–28]. The higher burden of health and social challenges present in the VA versus non-VA veteran population may be a consequence of the VA healthcare benefits eligibility criteria, which is based on each veteran’s military service history, disability rating, income level, and other benefits applicants receive (e.g., VA pension benefits).

Although prior research has provided insight into the sociodemographic and health characteristics that may vary between veterans who use the VA for their healthcare and non-VA veterans, at least two important gaps in the literature remain. First, most studies have focused on VA enrollees, a population that differs from veterans who use the VA for their healthcare [23–28], which is the patient population captured in the VA EHR. Veterans who use VA healthcare services live closer to VA facilities [29–32], and are more likely to have a psychiatric or substance use disorder diagnosis [29, 30], and greater healthcare needs [29, 31] than VA-enrolled veterans who do not. Because not all VA-enrolled veterans utilize VA health care services each year, prior research documenting sociodemographic and health differences in veterans by VA enrollment status may not adequately capture important differences between veterans overall and the VA patient population captured in the VA EHR. Second, the demographic profile of veterans, generally, and of the VA patient population in particular is changing: over the last two decades the age distribution has become younger, and the share of women veterans and racial/ethnic minorities has increased over the last two decades [33]; however, only two published studies have analyzed data that were collected within the past 10 years [26, 34], one of which limited its analysis to veterans with service-connected conditions [34, 35], and another that was focused on examining sociodemographic and health differences in veterans with versus without health coverage [26]. Therefore, the results of previous studies that have examined the differences between VA enrollees and non-VA veterans do not reflect the changing demographic profile of veterans, the representativeness of the VA patient population as contained in the VA EHR data remains unknown.

To address these limitations, we leveraged data from the 2019 National Health Interview Survey (NHIS) to characterize differences in the distribution of sociodemographic characteristics, physical and mental health, and health behaviors in US military veterans who did and did not use VA healthcare services during the past year. For this analysis, we selected variables that if they (a) have been previously shown to vary between VA and non-VA veterans or (b) are factors measured and available for study in the VA EHR data*.* The 2019 NHIS data are particularly well-suited for this analysis because of their large sample and ability to differentiate between veterans receiving VA healthcare services and veterans not receiving any past-year VA care. As such, this study provides the most current description of 1 year of VA use and non-use among non-institutionalized veterans.

## Methods

### Study population

We analyzed data on US veterans from the 2019 NHIS, a nationally representative household survey of the civilian noninstitutionalized US population. The investigation was carried out in accordance with the latest version of the Declaration of Helsinki and informed consent was obtained from all survey participants. The 2019 NHIS Sample Adult component included 31,997 adults, aged ≥18 years, of which 3061 (9.6%) were veterans, defined as adults who had ever served on active duty in the US Armed Forces, military Reserves, or National Guard and were not currently on active duty [36]. After excluding 10 respondents with missing age information, the analytic sample included 3051 veterans. These publicly available data are exempt from IRB review.

### Measures

#### Past year VA healthcare use

Our primary predictor variable was past-year use of VA healthcare services. This variable captures all participants whose data would be included in the VA EHR. Past year VA healthcare use was assessed using the question “*During the past 12 months, did you receive any care at a Veteran’s Health Administration facility or receive any other healthcare paid for by the VA?*” A dichotomous variable assessed whether veterans did or did not endorse past year use of VA healthcare services, regardless of whether they also utilized a different type of healthcare coverage (labeled hereafter as “VA patients” and “non-VA veterans”, respectively).

#### Sociodemographic characteristics

Sociodemographic variables for this study included age (18–34, 35–44, 45–54, 55–64, 65+), gender (male, female), ethnicity and race (Hispanic, non-Hispanic: White, Black, Asian or Pacific Islander, other [Native American, Alaska Native, Other Race]), sexual orientation (heterosexual, sexual minority), education level (<high school, high school or equivalent, some college or more), and family income relative to the federal poverty line (FPL; < 100% FPL, 100–199% FPL, 200–399% FPL, or ≥ 400% FPL).

#### Chronic health conditions

Participants reported whether a doctor or other healthcare professional had ever diagnosed them with high blood pressure, heart disease, diabetes, cancer (excluding non-melanoma skin cancer), arthritis, asthma, or chronic lung disease (i.e., chronic obstructive pulmonary disease, emphysema, or chronic bronchitis). In addition to considering the 7 chronic health conditions individually, we also created a composite variable, coded yes if a participant reported having ever being diagnosed with 1 or more of the 7 selected chronic conditions. Self-reported physician-diagnosed medical conditions have been found to have high validity [37].

#### Pain

Pain frequency, severity, and specific pain conditions were assessed using questions developed by the Washington Group on Disability Statistics [38]. Respondents were first asked “*In the past 3 months, how often did you have pain? Would you say never, some days, most days, or every day?*” For those who had pain at least some days, a follow-up question assessing bothersomeness was asked: “*Thinking about the last time you had pain, how much pain did you have—a little, between a little and a lot, or a lot*?” Participants who reported pain at least some days in the past 3 months were considered to have any pain. Participants who reported pain on “most days” or “every day” during the past 3 months were considered to have frequent pain. Participants who reported pain on “most days” or “every day” in the past 3 months *and* that the pain bothered them “a lot” were considered to have severe pain. Finally, participants were asked separate questions about pain in specific areas of the body (back; hands, arms, or shoulder; hips, knees, or feet; abdominal, pelvic, or genitals; migraines or headaches; and tooth or jaw) in the past 3 months, and whether they had symptoms of arthritis-related joint pain in the past 30 days. All pain measures have been extensively validated in the US and internationally [38].

#### Mental health status

Depressive symptom severity was assessed using the Patient Health Questionnaire—version 8 (PHQ-8), with a value of ≥10 used to identify adults experiencing depression [39]. Generalized Anxiety Disorder scale—version 7 (GAD-7) was used to assess anxiety, with moderate/severe anxiety symptoms indicated by GAD-7 scores ≥10 [40].

#### Combustible and electronic cigarette use

Participants were categorized into three mutually exclusive groups based on whether they had smoked ≥100 cigarettes in their lifetime and smoked at least some days in the past 30 days: current smokers (≥100 lifetime cigarettes and past 30-day use), former smokers (≥100 lifetime cigarettes and no past 30-day use), and never smokers (smoked < 100 cigarettes in their lifetime). Current electronic cigarette use or “vaping” was based on respondents endorsing they now use electronic cigarettes either every day or some days.

#### Self-reported health status, disability, and obesity

An indicator variable for fair or poor self-reported health was constructed based on responses to the question “*Would you say your health in general is excellent, very good, good, fair, or poor?*”; coded as 1 if a participant endorsed fair or poor health and coded as 0 if they endorsed excellent, very good, or good. This dichotomous measure is a reliable and valid measure of general physical well-being and highly correlated with objective measures of functional impairment, morbidity, and mortality [41, 42]. Disability was assessed using the Washington Group Composite Disability indicator. Participants who reported having serious difficulty in either seeing, hearing, mobility, communication, cognition, or self-care were classified as having a disability [38]. Obesity was defined as current body mass index ≥30 kg/m^2^ [43].

### Statistical analysis

Veterans were stratified by past-year VA healthcare use, and Pearson’s *χ*^2^ tests were used to evaluate differences between VA patients and non-VA veterans on sociodemographic characteristics, chronic health conditions, pain, mental health status, combustible cigarette use and vaping, and self-reported health. The *χ*^2^ test assumes the data were obtained through random selection, the data are frequencies or counts, with mutually exclusive levels of the variable, the study groups are independent, and the value of the cell expected should be 5 or more in at least 80% of the cells, with no cell having an expected count of less than one [44, 45]. In accordance with the American Statistical Association, we reported the actual *P* values, rather than expressing a statement of inequality (*P* < .05), to avoid the potential problem of incorrectly interpreting a *P* value as significant or not based on a pre-determined threshold value [46, 47]. All percentages and standard errors were calculated with SAS-callable SUDAAN 11.0.1 and NHIS sample weights were used to account for the complex survey design and survey nonresponse to produce estimates nationally representative of the non-institutionalized population of veterans residing in the US. Multivariable logistic regression models (SAS-callable SUDAAN 11.0.1) using sample weights generated weighted predicted marginal prevalence estimates (back-transformed from marginal log-odds) of sociodemographic and medical profiles in each US veteran group (VA patients and non-VA veterans), both unadjusted and adjusted for sociodemographic factors related to VA healthcare use, including age, gender, race and ethnicity, education, and family income. Predicted marginal prevalences were then used to calculate risk differences (RD) and adjusted risk differences (aRD) with 95% confidence intervals (CIs), which estimate group differences in absolute risk between VA patients and non-VA veterans. We estimated unadjusted and adjusted odds ratios (presented in the online appendix), which we transformed into Cohen’s *d* by $d={L}_{OR}\frac{\sqrt{3}}{\pi }$, where *π* =3.14159 and *L*_*OR*_ is the natural logarithm of the odds ratio to provide information on the magnitude of the effects [48], with effect sizes of *d* = 0.2, 0.5, and 0.8 indicating “small”, “medium”, and “large” effects, respectively [49].

## Results

Most veterans were male (89.2%), non-Hispanic White (79.0%), heterosexual (97.4%), completed some college or more (65.4%); 47.9% aged 65 and above and 45.1% reported a family income > 400% the federal poverty line (Table 1). Approximately 32% of veterans reported receiving past-year VA healthcare services. Non-VA veterans were more likely to have higher incomes and to be non-Hispanic White than VA patients.

**Table 1:** Sociodemographic characteristics of US military veterans overall and by past-year use of Veterans Administration (VA) healthcare: NHIS 2019

	All Veterans (***N*** = 3051)			Past Year VA Care^a^ (***N*** = 983)			No past year VA Care^b^ (***N*** = 2068)			***χ***^**2**^ (df), ***p***-value
	n	%	SE	n	%	SE	n	%	SE	
**Age Category**^c^										1.41 (4), 0.229
18–34	219	10.31	0.89	80	11.25	1.41	139	9.90	1.03	
35–44	233	9.29	0.67	81	9.33	1.08	152	9.27	0.80	
45–54	349	13.79	0.84	93	11.54	1.36	256	14.77	1.07	
55–64	526	18.72	0.80	161	17.32	1.36	365	19.33	1.02	
65+	1724	47.89	1.15	568	50.55	2.02	1156	46.73	1.34	
**Gender**										0.12 (1), 0.726
Male	2750	89.17	0.66	890	89.55	1.22	1860	89.01	0.83	
Female	301	10.83	0.66	93	10.45	1.22	208	10.99	0.83	
**Race and ethnicity**										8.66 (4), <.0001
Non-Hispanic White	2470	79.00	1.11	729	70.86	1.99	1741	82.56	1.22	
Non-Hispanic Black	315	11.48	0.82	153	18.05	1.67	162	8.61	0.87	
Non-Hispanic Asian	43	1.68	0.33	16	2.39	0.80	27	1.37	0.31	
Hispanic	147	5.11	0.50	62	6.56	0.88	85	4.47	0.62	
Other	76	2.73	0.44	23	2.14	0.50	53	2.99	0.58	
**Education level**^c^										0.54 (2), 0.584
Less than high school	155	5.79	0.52	56	6.67	1.04	99	5.40	0.62	
High school	795	28.82	1.03	274	28.67	1.77	521	28.88	1.23	
Some college or more	2084	65.39	1.11	650	64.66	1.87	1434	65.72	1.29	
**Family Income**^d^										13.75 (3), <.0001
< 100% FPL	161	5.11	0.51	61	6.07	0.92	100	4.68	0.60	
100% < =FPL < 200%	465	14.63	0.79	209	19.36	1.43	256	12.56	0.97	
200% < =FPL < 400%	1029	35.14	1.05	372	38.98	1.87	657	33.47	1.24	
> 400% FPL	1396	45.12	1.18	341	35.58	1.82	1055	49.29	1.42	
**Sexual orientation**^e^										0.63 (1), 0.438
Heterosexual	2964	97.42	0.36	947	97.00	0.60	2017	97.61	0.46	
Sexual minority	73	2.58	0.36	31	3.00	0.60	42	2.39	0.46	

*NHIS* National health interview survey, *SE* Standard error, *FPL* Federal poverty line, *df* Degrees of freedom

^a^Past-year VA care determined by respondents answering “yes” to the question “During the past 12 months, did you receive any care at a Veteran’s Health Administration facility or receive any other health care paid for by the VA?”

^b^No past-year VA care determined by respondents answering “no” to the question “During the past 12 months, did you receive any care at a Veteran’s Health Administration facility or receive any other health care paid for by the VA?”

^c^Total slightly less than total population N because 0.56% (*n* = 17) of respondents were missing data on education. Respondents who answered “something else” or “I don’t know the answer” Education data were missing for 17 participants who either refused to answer or reported “I don’t know” to NHIS questions on educational attainment

^d^Percentages will not necessarily add to 100 because of rounding

^e^Total slightly less than total population N because 0.46% (*n* = 14) of respondents were missing data on sexual orientation (e.g., answered “something else” or “I don’t know the answer” to survey questions on sexual orientation)

VA patients had a higher burden of any chronic health condition (aRD = 11.94; 95%CI = 8.08–15.80), high blood pressure (aRD = 12.46; 95%CI = 8.25–16.67), diabetes (aRD = 7.73; 95%CI = 4.31–11.15), arthritis (aRD = 15.17; 95%CI = 10.90–19.45), and chronic lung disease (aRD = 6.65; 95%CI = 3.84–9.45) than non-VA veterans (Table 2). Pain was more prevalent among VA patients than non-VA veterans. The highest differences in pain prevalence were for any pain (aRD = 13.02; 95%CI = 9.01–17.03), frequent pain (aRD = 19.89; 95%CI = 15.64–24.14) and severe pain (aRD = 7.78; 95%CI = 4.24–10.71). Both depressive symptoms (aRD = 5.70; 95%CI = 2.91–8.49) and anxiety symptoms (aRD = 6.72; 95%CI = 4.28–9.16) were also more prevalent in VA patients than non-VA veterans. Although group differences in current cigarette use and current electronic cigarette use were negligible, VA patients were more likely to be former smokers (aRD = 8.73; 95%CI = 4.55–12.92) than non-VA veterans. Moderate differences in overall measures of general health (aRD = 12.87; 95%CI = 9.18–16.55) and disability (aRD = 10.58; 95%CI = 7.21–13.94) were observed between the two groups, with 27.9% of VA patients reporting fair or poor health and 20.2% reporting disability, compared to 18.0 and 11% of non-VA veterans.

**Table 2:** Health conditions and behaviors in US military veterans by past-year use of VA health care: NHIS, 2019

Health Condition	Past-year VA Care^a^	No past-year VA care^b^	Comparison of past-year VA care^a^ to no VA care^b^			
	% (SE)	% (SE)	RD^c^ (95% CI)	Cohen’s ***d***	aRD^c,d^ (95% CI)	Cohen’s ***d***
**Chronic Health Conditions**						
High Blood Pressure	57.59 (1.74)	47.11 (1.21)	10.48 (6.69, 14.26)	0.28	12.46 (8.25, 16.67)	0.28
Heart Disease^e^	21.80 (1.68)	19.24 (0.93)	2.56 (−0.98, 6.11)	0.10	4.11 (0.47, 7.74)	0.14
Diabetes	24.87 (1.53)	18.98 (1.02)	5.89 (2.08, 9.71)	0.26	7.73 (4.31, 11.15)	0.30
Cancer^f^	17.34 (1.45)	16.50 (0.89)	0.84 (−2.40, 4.08)	0.04	0.53 (− 2.74, 3.80)	0.02
Arthritis	45.94 (1.89)	31.60 (1.21)	14.34 (10.09, 18.59)	0.37	15.17 (10.90, 19.45)	0.36
Asthma	13.32 (1.27)	10.98 (0.91)	2.33 (− 0.86, 5.53)	0.12	2.66 (−0.57, 5.88)	0.14
Chronic lung disease	13.50 (1.13)	7.96 (0.70)	5.54 (2.94, 8.13)	0.35	6.65 (3.84, 9.45)	0.38
Any Chronic Health Condition	80.56 (1.40)	69.43 (1.24)	11.13 (7.65, 14.61)	0.41	11.94 (8.08, 15.80)	0.36
**Pain Conditions, past 3 months**^g^						
Any pain, past 3 months	78.87 (1.57)	65.86 (1.33)	13.02 (9.01, 17.03)	0.37	12.53 (8.44, 16.62)	0.35
Back pain, past 3 months	59.68 (1.87)	42.59 (1.44)	17.08 (12.53, 21.63)	0.39	15.80 (11.20, 20.41)	0.35
Hands, arms, or shoulders, past 3 months	50.65 (1.96)	34.87 (1.36)	15.79 (11.30, 20.27)	0.36	14.96 (10.57, 19.35)	0.34
Hips, knees, or feet, past 3 months	56.43 (1.92)	42.15 (1.36)	14.28 (9.85, 18.70)	0.32	14.50 (10.08, 18.91)	0.32
Abdominal, pelvic, or genital pain, past 3 months	14.69 (1.35)	7.60 (0.69)	7.09 (4.16, 10.02)	0.41	7.08 (4.19, 9.96)	0.41
Migraines or headaches, past 3 months	25.61 (1.80)	15.29 (1.04)	10.32 (6.41, 14.23)	0.38	10.28 (6.33, 14.23)	0.35
Tooth or jaw pain, past 3 months	11.50 (1.10)	8.61 (0.84)	2.89 (0.39, 5.39)	0.18	3.20 (0.61, 5.79)	0.19
Arthritis related joint pain, past 30 days	35.99 (1.85)	23.06 (1.07)	12.93 (8.76, 17.09)	0.37	13.12 (8.87, 17.36)	0.35
**Pain frequency, past 3 months**^g^						
Frequent pain (Most or every day)	45.31 (1.95)	25.43 (1.11)	19.89 (15.64, 24.14)	0.51	19.87 (15.64, 24.10)	0.49
**Pain severity during the last time you had pain**						
More than little pain	53.81 (1.87)	33.15 (1.28)	20.66 (16.47, 24.85)	0.48	21.21 (16.93, 25.49)	0.48
A lot of pain	17.21 (1.39)	9.73 (0.83)	7.48 (4.24, 10.71)	0.37	8.45 (5.21, 11.70)	0.40
**Mental Health Conditions**						
Moderate/ Severe depressive symptoms (PHQ)^h^	11.60 (1.19)	5.90 (0.75)	5.70 (2.91, 8.49)	0.43	6.72 (3.86, 9.58)	0.47
Moderate/severe anxiety symptoms^i^	10.82 (1.09)	4.10 (0.52)	6.72 (4.28, 9.16)	0.69	6.90 (4.56, 9.24)	0.67
**Combustible cigarette use and e-cigarette use**						
Current Smoker	17.54 (1.38)	17.18 (1.22)	0.36 (−3.15, 3.87)	0.02	0.79 (−2.81, 4.39)	0.03
Former Smoker	49.03 (1.76)	39.53 (1.25)	9.50 (5.45, 13.54)	0.23	8.73 (4.55, 12.92)	0.20
Current e-cig use^j^	4.44 (0.86)	4.55 (0.60)	−0.11 (−2.17, 1.94)	−0.02	−0.74 (− 2.79, 1.31)	−0.10
**Health Status**						
Fair or Poor Health	27.88 (1.53)	17.95 (1.06)	9.93 (6.34, 13.51)	0.34	12.87 (9.18, 16.55)	0.40
Disability^k^	20.16 (1.51)	11.07 (0.77)	9.09 (5.71, 12.47)	0.42	10.58 (7.21, 13.94)	0.44
Obese	38.11 (1.75)	33.46 (1.22)	4.65 (0.47, 8.83)	0.11	4.26 (0.15, 8.36)	0.10

*NHIS* National Health Interview Survey, *RD* Risk Difference, *CI* Confidence Interval

^a^Past-year VA care determined by respondents answering “yes” to the question “During the past 12 months, did you receive any care at a Veteran’s Health Administration facility or receive any other health care paid for by the VA?”

^b^No past-year VA care determined by respondents answering “no” to the question “During the past 12 months, did you receive any care at a Veteran’s Health Administration facility or receive any other health care paid for by the VA?”

^c^Logistic models were used to generate predicted marginal prevalences which are standardized to the distribution of sociodemographic characteristics of the sample. Risk differences (RD) indicate group differences in absolute risk

^d^Regressions adjusted for age category (18–34; 35–44; 45–54; 55–64; 65+), gender (male/female), race/ethnicity (non-Hispanic White, non-Hispanic Black, Hispanic, other), education (less than high school, high school or equivalent; some college or more), poverty status based on Federal Poverty Level (FPL) (< 100% FPL; 100% < =FPL < 200%; 200% < =FPL < 400%; > 400% FPL)

^e^Doctor ever told them that they had coronary heart disease, angina pectoris, heart attack, or stroke

^f^Doctor ever told them they had cancer, excluding non-melanoma skin cancer

^g^In the past three months, how often did you have pain? Pain questions were asked to those with response of some days, most days, or every day

^h^Moderate to severe depressive symptoms based on PHQ8 score of above 9

^i^Moderate to severe anxiety symptoms based on GAD7 score of above 9

^j^Use e-cigarettes or other electronic vaping products every or somedays

^k^Based on the Washington Group Short Set Composite Disability Indicator. Respondent endorsing vision problems, use of a hearing aid, difficulty climbing steps, difficulty communicating, difficulty with self-care, or difficulty remembering or concentrating

**Fig. 1:**
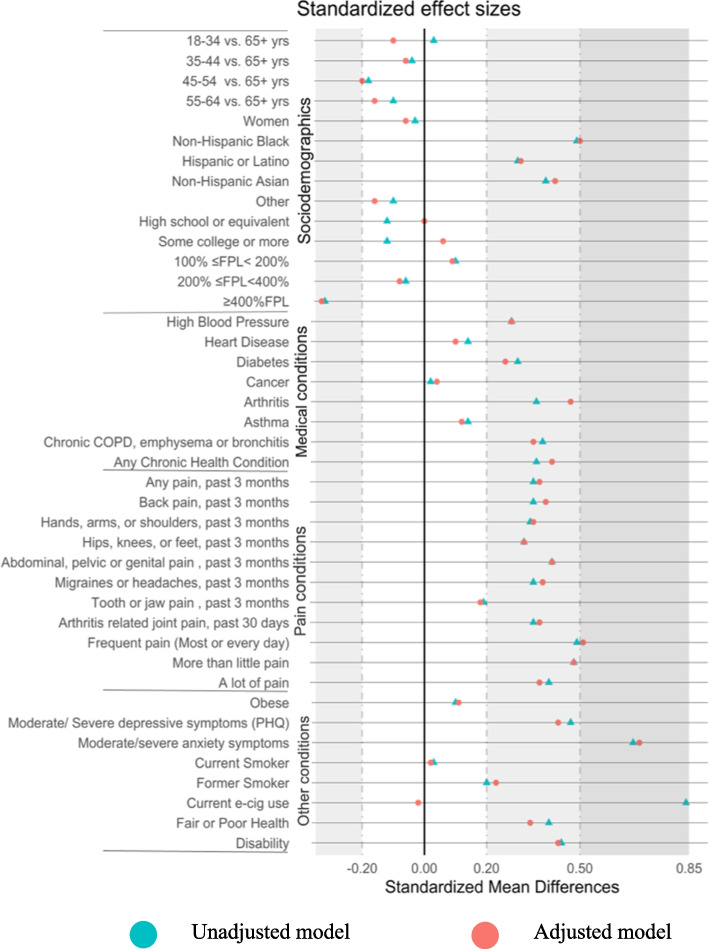
Love plot displaying unadjusted and adjusted effect sizes of 31 characteristics between US military veterans with past-year VA care and veterans without past-year VA care in the National Health Interview Survey (NHIS), 2019. Logistic models were used to generate unadjusted predicted marginal prevalences and adjusted predicted marginal prevalences, standardized to the distribution of sociodemographic characteristics of the sample. Regressions adjusted for age category, gender, race/ethnicity, education, and poverty status based on Federal Poverty Level. The unstandardized regression coefficients and pooled variance from the unadjusted and adjusted regression models were then used to calculate the Cohen’s *d*

Figure 1 shows the magnitude of the sociodemographic and health differences between VA patients and non-VA veterans. The difference between VA patients and non-VA veterans in the prevalence of non-Hispanic Blacks were moderate (*d* ≥ 0.50), although differences between VA patients and non-VA veterans were small for all other race and ethnicity groups and income. For health conditions, we observed the largest group differences between VA patients and non-VA veterans for frequent pain (*d* = 0.49), severe pain (*d* = 0.40), depressive symptoms (*d* = 0.67), and anxiety symptoms (*d* = 0.47).

## Discussion

Using data from the nationally representative 2019 National Health Interview Survey, we documented important differences in the distribution of socioeconomic and health characteristics of veterans who use and who do not use VA services. There were several important findings from this study. First, consistent with prior work, the sociodemographic composition of VA patients in 2019 differed from non-VA veterans, the primary population of interest for many VA EHR-based studies [23]. Our finding that members of disadvantaged racial and ethnic minority groups and low-income veterans were overrepresented in the VA patient population is consistent with the prior research that defined VA healthcare use by VA enrollment status rather than VA healthcare use [23–27]. However, we observed relatively minimal differences in the age and gender distribution of VA patients and non-VA veterans, in contrast to these previously published studies that found women and younger veterans overrepresented in VA enrollees relative to non-enrolled veterans [23–27]. Although we cannot explain why VA enrollees, but not VA patients, are more likely to be younger and female than other veterans, women and younger VA enrollees may prefer to receive their care outside of the VA system; these groups may have greater access to non-VA healthcare (e.g., as part of their employment benefits) or have better health and lower healthcare needs than their peers.

Second, VA patients were disproportionately burdened by physical and psychological morbidity and disability, including higher prevalences of high blood pressure, diabetes, arthritis, chronic lung disease, frequent and severe pain, depression and anxiety symptoms, and fair/poor self-reported health. Although the over-representation of high-risk health conditions may be expected in a patient population accessing outpatient medical and hospital services [24, 27], the over-representation of physical and psychological morbidity and disability in the VA patient population may be exacerbated by the eligibility criteria for VA services, which prioritizes veterans with severe income limitations and service-connected disability [34, 35]. Veterans with service-connected conditions, particularly those with psychiatric disorders such as depression and PTSD, depend heavily upon the VA for health care. For example, Maynard et al. [34] found that veterans with service-connected psychiatric disorders accounted for most hospitalizations in the VA system, and almost half of VA enrollees with PTSD and/or major depression had one or more mental health visits in 2016. As such, we would expect VA patients to have greater physical and psychological morbidity and disability than non-VA veterans.

The over-representation of high-risk sociodemographic and health conditions in the VA patient population indicate that VA EHR-based studies may yield estimates that are not generalizable to the overall veteran population. However, statistical methods have been proposed to improve the generalizability of EHR results to populations of clinical and policy interest [50, 51]. For example, the substantial body of literature on suicide and its potential causes among veterans has relied heavily on data from the VA’s EHR databases [13, 52, 53], which will result in gaps in knowledge about those who do not receive care within the VA. Using information on the differences in the distribution of socioeconomic and health characteristics of veterans who use and who do not use VA services, future VA EHR-based studies could apply selection probabilities with model-based standardizations to estimate the results in the total US veteran population. The same approach can be applied to estimate the treatment effect in a population distinct from the study sample. For example, given that the factors that determine whether a person receives healthcare through the VA versus Medicare are well documented [54–56], similar methods could be applied to generalize results from the VA EHR data to the Medicare population to estimate the expected effect, for example, of implementing a VA program in the Medicare population. Although the specific set of characteristics that must be included in a selection model will depend on the research question being investigated, our study provides critical information on the variables that differentiate VA patients from non-VA veterans that future studies require to accurately estimate the conditional probability of being selected.

Study limitations are noted. First, the NHIS sample does not include homeless individuals or those in institutional settings. Although homeless veterans are disproportionately affected by physical and mental illness, they compromise a very small fraction of the VA patient population (~ 37,000 veterans were homeless in 2020 [57]), and thus their effect on the overall findings would be limited. Second, self-report measures of chronic medical conditions, mental distress, and anxiety in the NHIS are not confirmed with medical diagnosis or collateral information. Social desirability could lead to underreporting of stigmatized conditions, although there is no reason to believe this would vary by past-year VA healthcare use. Third, given the documented changes in the underlying VA patient population over time, our findings may not generalize to earlier years. Fourth, neither VA patients nor non-VA veterans were engaged as stakeholder partners in the planning, conduct, or dissemination phases of this study. However, our research team was comprised of a diverse set of experts, including VA research scientists and VA clinician/researchers, which are considered patient partners and stakeholder partners by the PCORI Engagement Rubric [58].

Our study provides valuable results on the representativeness of the VA patient population to the overall US veteran population. These findings will be useful for both hypothesizing about how inferences derived from VA EHR data will generalize to the overall US veteran population and minimizing the effect of bias in the context of differential patient population selection that affect both exposures and outcomes. Differences between the VA patient population and overall US veteran population should be continuously monitored to identify potential influential changes in their sociodemographic and clinical profile over time. Future research should investigate how to best use VA EHR data to better understand and meet the needs of all US veterans, including VA enrollees who might leave the VA for other public insurance options (e.g., Medicaid, Medicare) or those who choose community providers.

## Supplementary Information

**Additional file 1: Appendix Table 1.** Health conditions and behaviors in US military veterans by past-year use of VA health care: NHIS, 2019.

## Data Availability

The datasets generated and analyzed during the current study are publicly available in the National Health Interview Survey, 2019 at https://www.cdc.gov/nchs/nhis/2019nhis.htm.
