# Supplementary material for: Comparing mental and physical health of U.S. veterans by VA healthcare use: implications for generalizability of research in the VA electronic health records
Source: BMC Health Serv Res. 2022 Dec 9;22:1500. doi: 10.1186/s12913-022-08899-y (PMC9733218; doi:10.1186/s12913-022-08899-y)
Supplement: Supplementary file 1 — Additional file 1: Appendix Table 1. Health conditions and behaviors in US military veterans by past-year use of VA health care: NHIS, 2019. [file 12913_2022_8899_MOESM1_ESM.docx]

**Appendix Table 1.** Health conditions and behaviors in US military veterans by past-year use of VA health care: NHIS, 2019

|  | **Past 12-month VA Care** | **No VA care** | **Comparison of VA care to no VA care** | | | | |
| --- | --- | --- | --- | --- | --- | --- | --- |
| **Health Condition** | **% (SE)** | **% (SE)** | **OR (95% CI)** | **Cohen’s D** | **aOR (95% CI)** | **Cohen’s D** |  |
| **Chronic Health Conditions** |  |  |  |  |  |  |  |
| High Blood Pressure | 57.59 (1.74) | 47.11 (1.21) | 1.65 (1.38, 1.99) | 0.28 | 1.65 (1.39, 1.96) | 0.28 |  |
| Heart Disease^c^ | 21.80 (1.68) | 19.24 (0.93) | 1.20 (0.94, 1.53) | 0.10 | 1.28 (1.04, 1.59) | 0.14 |  |
| Diabetes | 24.87 (1.53) | 18.98 (1.02) | 1.59 (1.25, 2.03) | 0.26 | 1.73 (1.37, 2.19) | 0.30 |  |
| Cancer^d^ | 17.34 (1.45) | 16.50 (0.89) | 1.07 (0.83, 1.38) | 0.04 | 1.04 (0.82, 1.31) | 0.02 |  |
| Arthritis | 45.94 (1.89) | 31.60 (1.21) | 1.94 (1.60, 2.36) | 0.37 | 1.91 (1.59, 2.28) | 0.36 |  |
| Asthma | 13.32 (1.27) | 10.98 (0.91) | 1.25 (0.93, 1.69) | 0.12 | 1.28 (0.95, 1.72) | 0.14 |  |
| Chronic COPD, emphysema, or bronchitis | 13.50 (1.13) | 7.96 (0.70) | 1.87 (1.41, 2.48) | 0.35 | 2.01 (1.52, 2.66) | 0.38 |  |
| Any Chronic Health Condition | 80.56 (1.40) | 69.43 (1.24) | 2.09 (1.63, 2.68) | 0.41 | 1.91 (1.53, 2.39) | 0.36 |  |
| **Pain Conditions, past 3 months^e^** |  |  |  |  |  |  |  |
| Any pain, past 3 months | 78.87 (1.57) | 65.86 (1.33) | 1.96 (1.57, 2.44) | 0.37 | 1.89 (1.51, 2.35) | 0.35 |  |
| Back pain, past 3 months | 59.68 (1.87) | 42.59 (1.44) | 2.02 (1.67, 2.45) | 0.39 | 1.89 (1.57, 2.29) | 0.35 |  |
| Hands, arms, or shoulders, past 3 months | 50.65 (1.96) | 34.87 (1.36) | 1.93 (1.60, 2.33) | 0.36 | 1.85 (1.55, 2.22) | 0.34 |  |
| Hips, knees, or feet, past 3 months | 56.43 (1.92) | 42.15 (1.36) | 1.79 (1.49, 2.15) | 0.32 | 1.79 (1.50, 2.15) | 0.32 |  |
| Abdominal, pelvic or genital pain, past 3 months | 14.69 (1.35) | 7.60 (0.69) | 2.12 (1.59, 2.83) | 0.41 | 2.10 (1.59, 2.76) | 0.41 |  |
| Migraines or headaches, past 3 months | 25.61 (1.80) | 15.29 (1.04) | 2.00 (1.56, 2.57) | 0.38 | 1.90 (1.51, 2.39) | 0.35 |  |
| Tooth or jaw pain, past 3 months | 11.50 (1.10) | 8.61 (0.84) | 1.39 (1.05, 1.85) | 0.18 | 1.42 (1.08, 1.88) | 0.19 |  |
| Arthritis related joint pain, past 30 days | 35.99 (1.85) | 23.06 (1.07) | 1.95 (1.58, 2.40) | 0.37 | 1.90 (1.55, 2.32) | 0.35 |  |
| **Pain frequency, past 3 months** |  |  |  |  |  |  |  |
| Frequent pain (Most or every day) | 45.31 (1.95) | 25.43 (1.11) | 2.50 (2.07, 3.03) | 0.51 | 2.43 (2.02, 2.93) | 0.49 |  |
| **How much pain during the last time you had pain** |  |  |  |  |  |  |  |
| More than little pain | 53.81 (1.87) | 33.15 (1.28) | 2.40 (2.00, 2.86) | 0.48 | 2.40 (2.01, 2.88) | 0.48 |  |
| A lot of pain | 17.21 (1.39) | 9.73 (0.83) | 1.96 (1.48, 2.58) | 0.37 | 2.08 (1.59, 2.73) | 0.40 |  |
| **Other Health Conditions** |  |  |  |  |  |  |  |
| Obese | 38.11 (1.75) | 33.46 (1.22) | 1.23 (1.02, 1.49) | 0.11 | 1.20 (1.01, 1.44) | 0.10 |  |
| Moderate/ Severe depressive symptoms (PHQ)^f^ | 11.60 (1.19) | 5.90 (0.75) | 2.17 (1.50, 3.13) | 0.43 | 2.34 (1.65, 3.31) | 0.47 |  |
| Moderate/severe anxiety symptoms^g^ | 10.82 (1.09) | 4.10 (0.52) | 3.49 (2.36, 5.16) | 0.69 | 3.35 (2.30, 4.89) | 0.67 |  |
| **Health Behaviors** |  |  |  |  |  |  |  |
| Current Smoker | 17.54 (1.38) | 17.18 (1.22) | 1.03 (0.78, 1.35) | 0.02 | 1.06 (0.82, 1.36) | 0.03 |  |
| Former Smoker | 49.03 (1.76) | 39.53 (1.25) | 1.52 (1.27, 1.82) | 0.23 | 1.43 (1.20, 1.69) | 0.20 |  |
| Current e-cig use^h^ | 4.44 (0.86) | 4.55 (0.60) | 0.97 (0.58, 1.64) | -0.02 | 0.84 (0.50, 1.39) | -0.10 |  |
| **Health Status** |  |  |  |  |  |  |  |
| Fair or Poor Health | 27.88 (1.53) | 17.95 (1.06) | 1.85 (1.49, 2.31) | 0.34 | 2.06 (1.68, 2.53) | 0.40 |  |
| Disability^i^ | 20.16 (1.51) | 11.07 (0.77) | 2.16 (1.66, 2.82) | 0.42 | 2.24 (1.77, 2.85) | 0.44 |  |
| OR = Odds Ratio; CI = Confidence Interval; RR = Risk Ratio. ^a^ Logistic models were used to generate predicted marginal prevalences which are standardized to the distribution of sociodemographic characteristics of the sample. Odds ratios (OR) indicate group differences in odds, risk ratios (RR) indicate group differences in relative risk, and risk differences (RD) indicate group differences in absolute risk. ^b^ Regressions adjusted for age category (18-34; 35-44; 45-54; 55-64; 65+), sex (male/female), race/ethnicity (non-Hispanic White, non-Hispanic Black, Hispanic, other), education (less than high school, high school or equivalent; some college or more), poverty status based on Federal Poverty Level (FPL) (<100% FPL; 100% <=FPL<200%; 200% <=FPL<400%; >400% FPL ) ^c^ Doctor ever told them that they had coronary heart disease, angina pectoris, heart attack, or stroke ^d^ Doctor ever told them they had cancer, excluding non-melanoma skin cancer ^e^ In the past three months, how often did you have pain? Pain questions were asked to those with response of some days, most days, or every day. ^f^ Moderate to severe depressive symptoms based on PHQ9 score of above 9 ^g^ Moderate to severe anxiety symptoms based on GAD9 score of above 9 ^h^ Use e-cigarettes or other electronic vaping products every or somedays ^i^ Based on the Washington Group Short Set Composite Disability Indicator. Respondent endorsing vision problems, use of a hearing aid, difficulty climbing steps, difficulty communicating, difficulty with self-care, or difficulty remembering or concentrating. | | | | | | | |
